# Supplementary figures and images for: Lys417 acts as a molecular switch that regulates the conformation of SARS-CoV-2 spike protein
Source: eLife. 2023 Nov 22;12:e74060. doi: 10.7554/eLife.74060 (PMC10695562; doi:10.7554/eLife.74060)

## Slide 1
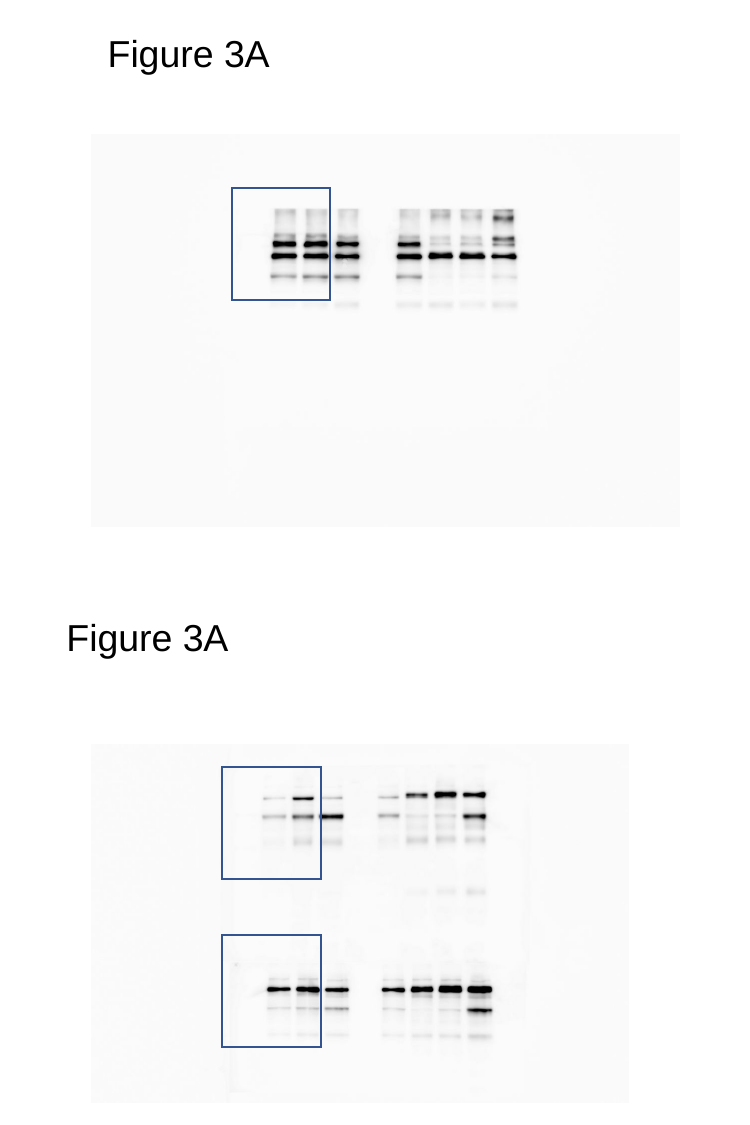

Figure 3A
Figure 3A

Supplement: Figure 3—source data 1. [file elife-74060-fig3-data1.pptx]

## Slide 1
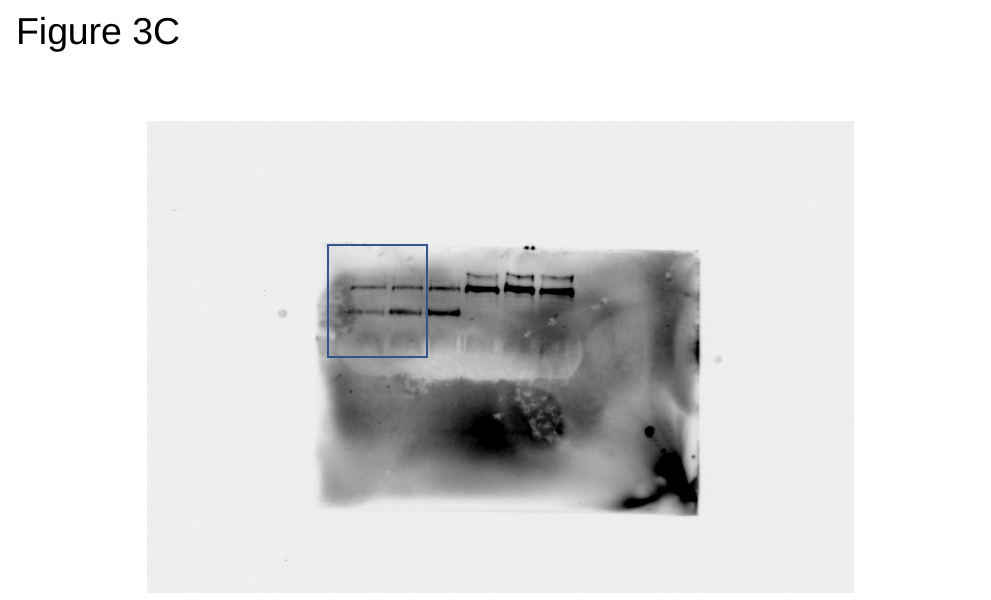

Figure 3C

Supplement: Figure 3—source data 3. [file elife-74060-fig3-data3.pptx]
