# Supplementary material for: Lys417 acts as a molecular switch that regulates the conformation of SARS-CoV-2 spike protein
Source: eLife. 2023 Nov 22;12:e74060. doi: 10.7554/eLife.74060 (PMC10695562; doi:10.7554/eLife.74060)
Supplement: Supplementary file 1. [file elife-74060-supp1.docx]

**Table S1: Cryo-EM data collection, refinement and validation statistics.**

|  | K417-spike Closed  (EMD-42589)  (PDB 8UUL) | K417-spike  Open  (EMD-42590)  (PDB 8UUM) | V417-spike  Closed  (EMD-42591)  (PDB 8UUN ) | V417-spike  Open  (EMD-42592)  (PDB 8UUO) |
| --- | --- | --- | --- | --- |
| **Data collection**  **and processing** |  |  |  |  |
| Magnification | 130,000 | 130,000 | 130,000 | 130,000 |
| Voltage (kV) | 300 | 300 | 300 | 300 |
| Electron exposure (e–/Å^2^) | 40.00 | 40.00 | 40.00 | 40.00 |
| Defocus range (μm) | 0.8–2.4 | 0.8–2.4 | 0.8–2.4 | 0.8–2.4 |
| Pixel size (Å) | 0.664 | 0.664 | 0.664 | 0.664 |
| Symmetry imposed | C3 | C1 | C3 | C1 |
| Initial particle images (no.) | 57,795 | 57,795 | 45,999 | 45,999 |
| Final particle images (no.) | 23,665 | 19,305 | 9,608 | 19,988 |
| Map resolution (Å)  FSC threshold | 3.2  0.143 | 3.9 | 3.8  0.143 | 3.9 |
|  |  | 0.143 |  | 0.143 |
| Map resolution range (Å) | 3.0-7.0 | 3.6-8.4 | 3.6–8.4 | 3.6-8.4 |
|  |  |  |  |  |
| **Refinement** |  |  |  |  |
| Initial model used (PDB code) | 7TGY | 7TGX | 7TGY | 7TGX |
| Model resolution (Å)  FSC threshold | 3.45  0.5 | 4.16 | 3.96  0.5 | 4.2 |
|  |  | 0.5 |  | 0.5 |
| Map sharpening *B* factor (Å^2^) | -83.9 | -72.1 | -72.2 | -69.6 |
| Model composition  Non-hydrogen atoms  Protein residues  Ligands | 25891  3254  33 |  | 25829  3254  29 |  |
|  |  | 25786 |  | 25465 |
|  |  | 3247 |  | 3218 |
|  |  | 30 |  | 22 |
| *B* factors (Å^2^)  Protein  Ligand | 164.80  164.32 |  | 170.77  166.71 |  |
|  |  | 217.46 |  | 234.68 |
|  |  | 203.04 |  | 194.65 |
| R.m.s. deviations  Bond lengths (Å)  Bond angles (°) | 0.009  1.016 |  | 0.009  1.051 |  |
|  |  | 0.008 |  | 0.007 |
|  |  | 1.016 |  | 1.044 |
| Validation  MolProbity score  Clashscore  Poor rotamers (%) | 1.51  3.74  0.04 |  | 1.56  4.41  0.00 |  |
|  |  | 1.66 |  | 1.70 |
|  |  | 5.40 |  | 6.48 |
|  |  | 0.11 |  | 0.04 |
| Ramachandran plot  Favored (%)  Allowed (%)  Disallowed (%) | 95.00  5.00  0.00 |  | 95.09  4.91  0.00 |  |
|  |  | 94.58 |  | 95.06 |
|  |  | 5.42 |  | 4.94 |
|  |  | 0.00 |  | 0.00 |
